# FishFeats: streamlined quantification of multimodal labeling at the single-cell level in 3D tissues

**DOI:** 10.1093/bioinformatics/btag105

**Published:** 2026-03-02

**Authors:** Gaëlle Letort, Tanya Foley, Ilona Mignerey, Laure Bally-Cuif, Nicolas Dray

**Affiliations:** Dynamics of Developmental Decisions Unit, Institut Pasteur—Université Paris Cité, UMR3738, CNRS, Paris 75015, France; Zebrafish Neurogenetics Unit, Institut Pasteur—Université Paris Cité, UMR3738, CNRS, Paris 75015, France; Zebrafish Neurogenetics Unit, Institut Pasteur—Université Paris Cité, UMR3738, CNRS, Paris 75015, France; Zebrafish Neurogenetics Unit, Institut Pasteur—Université Paris Cité, UMR3738, CNRS, Paris 75015, France; Zebrafish Neurogenetics Unit, Institut Pasteur—Université Paris Cité, UMR3738, CNRS, Paris 75015, France

## Abstract

**Summary:**

Characterizing the distribution of biological marker expression at the single cell level in whole tissues requires diverse image analysis steps, such as segmentation of cells and nuclei, detection of RNA transcripts (or other staining), or their mapping (e.g. assigning nuclei/RNA dots to their corresponding cell). Several software programs or algorithms have been developed for each step independently but integrating them into a comprehensive pipeline for the quantification of individual cells from 3D imaging samples remains a significant challenge. We developed FishFeats, an open-source and flexible napari plugin, to perform all these steps together within the same framework, taking advantage of available and efficient software applications. The primary core of our pipeline is to propose a user-friendly tool for users who do not have a computational background. FishFeats streamlines extracting quantitative information from multimodal 3D fluorescent microscopy images (smFISH expression in individual cells, immunohistochemical staining, cell morphologies, cell classification) to a unified “cell-by-cell” table for downstream analysis, without requiring any coding. Our second focus is to propose and ease manual correction of each step to further improve accuracy, which can be critical for many biological studies.

**Availability and implementation:**

FishFeats is open source under the BSD-3 license, freely available on github: https://github.com/gletort/FishFeats (DOI 10.5281/zenodo.17701225). FishFeats is developed in python, as a napari plugin for the user interface. Documentation is available in the github pages: https://gletort.github.io/FishFeats/. To report an issue using FishFeats or contributing to it please file an issue in the github repository https://github.com/gletort/FishFeats/issues.

## 1 Introduction

Recently, research in biology increasingly focuses on single-cell resolution (Chen *et al.* 2025) and tremendous efforts have been placed on developing experimental set-ups to link cellular transcriptomics with cell morphology, spatial position within a tissue, and other cellular features, all at the single cell level. Numerous molecular biology techniques have been developed to enable multiplexed in situ labeling of cells, embryos, or tissues. These approaches often combine methods like single-molecule RNA visualization (e.g. smFISH) and protein detection (e.g. immunohistochemistry, or IHC) to simultaneously observe RNA transcripts alongside membrane, nuclear, or cytoplasmic components. However, extracting quantitative information from the acquired microscopy 3D images involves several image analysis steps. The number and efficiency of available tools to perform each of these image analysis steps are increasing but with it also the complexity of usage of these tools (Schlaeppi *et al.* 2022, Li *et al.* 2023, Nogare *et al.* 2023, Pylvänäinen *et al.* 2025). This makes it difficult for users who do not have a computational background to take advantage of these tools and perform their own analysis, especially if it requires combining them.

**Figure 1:**
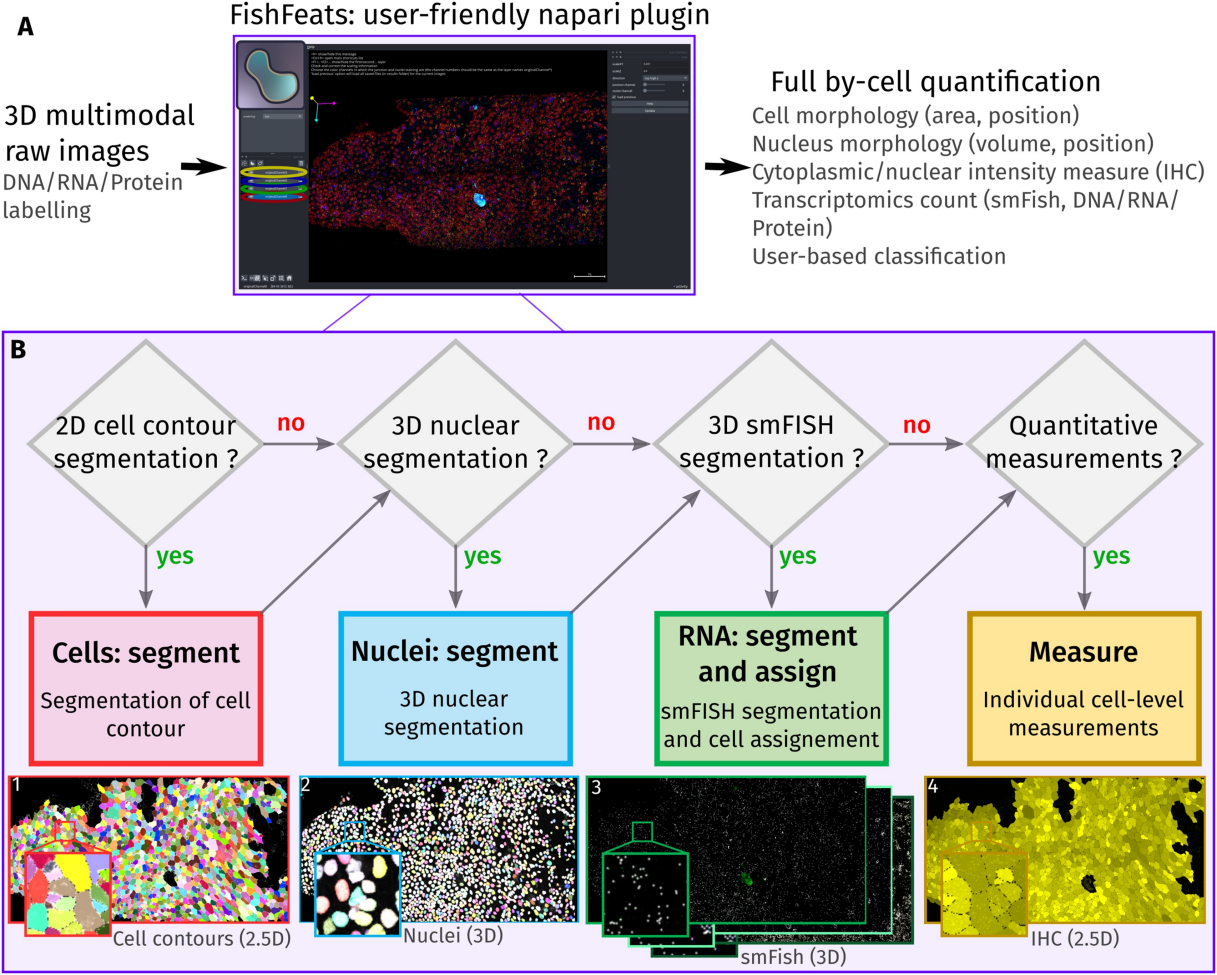
(A) The main FishFeats user interface is shown in the center. FishFeats is built on napari interface, with the image displayed in the center of the interface. The left panel contains the layers (here, the three 3D color channels) at the bottom and the display options for each layer above. The FishFeats with all the options and algorithm parameters are available on the right side. A specific help message for each option can be overlaid on the main window. The single output table is updated from this interface at each step. (B) Depending on the analysis, the user will follow a specific workflow, as described here. For each main step of the pipeline, snapshots of the results are shown, with the data dimensionality indicated in parentheses. 2.5D refers to 2D labels with 3D coordinates. The biological image shown in this figure corresponds to a dorsal view of a dissected zebrafish embryo brain, 4 months post-fertilization.

We present here our open-source and user-friendly pipeline, FishFeats (for Fluorescent in Situ Hybridization & Features), to facilitate the joint analysis of multiple quantitative cellular features (mRNA transcripts, IHC markers, morphometric measures) in 2D/3D at the single cell level in epithelia (Fig. 1A). FishFeats proposes a single interface to run several state-of-the-art algorithms as well as its own specific algorithms. Importantly, all quantifications are unified into a single table “per-cell,” suitable for downstream analysis. We strongly focused on its user friendliness through its graphical interface (Fig. 1A) and dedicated documentation, which are important aspects for the tool accessibility (Jamali *et al.* 2022, Pylvänäinen *et al.* 2025). The frequent feedback from its users was fundamental to identify and correct usability bottlenecks (Soltwedel and Haase 2024, Pylvänäinen *et al.* 2025). Particularly, one crucial component in all our biological applications was the possibility for manual correction. This is a tedious task that we now assist with different visualization options, dedicated shortcuts, or automated functions.

## 2 FishFeats

We chose to implement our pipeline as a napari plugin (Sofroniew *et al.* 2025) which is open-source, Python based, compatible with most image formats and, importantly for our purpose, has multiple visualization advantages (2D/3D, multilayered) allowing for more interactivity. Several steps are proposed (Supplementary 2, available as supplementary data at *Bioinformatics* online) as independently as possible for flexibility in the analysis. In most analyses, the first step consists in segmenting cells within a tissue. In developmental biology, cells are routinely identified by an apical marker of cellular junctions approximating a 2D sheet within a 3D image. FishFeats first does a projection and 2D segmentation of the cell contours and then places these labels in 3D (Fig. 1B). The projection can be calculated within the plugin or by an external software (LocalZProjector, CARE) (Weigert *et al.* 2018, Herbert *et al.* 2021). FishFeats offers an interface to perform the segmentation of these epithelium-like projections with Cellpose and Epyseg (Aigouy *et al.* 2020, Stringer *et al.* 2021, Pachitariu *et al.* 2025). Manual correction of the results is facilitated by napari Label editing tools and additional specific options such as keyboard shortcuts to switch channels on/off, merge cells, etc. (Supplementary 2.1.2, available as supplementary data at *Bioinformatics* online). The 2D cells are then re-positioned in 3D by assigning to each cell its most likely Z-position, which can be fine-tuned or manually corrected (Supplementary 2.2, available as supplementary data at *Bioinformatics* online). If the acquired images contain nuclear staining (or any structured labeling, such as vesicles), 3D segmentation can be done (Supplementary 2.3, available as supplementary data at *Bioinformatics* online) using Stardist or CellPose (Weigert *et al.* 2020, Stringer *et al.* 2021) (Fig. 1B), along with the possibility to filter and correct the results. Each segmented nucleus or object can then be paired with the apical cell surface it likely belongs to with the Hungarian algorithm (Kuhn 1955) (Supplementary 2.4, available as supplementary data at *Bioinformatics* online). Manual corrections of the cell-nucleus or cell-object association are made easy by clicking on the image with keyboard shortcuts (Supplementary 2.4, available as supplementary data at *Bioinformatics* online).

A core development of the pipeline is the feature to detect and assign single RNA molecules (visible as dots) to their corresponding cell. Segmentation of RNA dots is performed in 3D (Fig. 1B) with Big-Fish (Imbert *et al.* 2021), a robust spot detection library. Important parameters of the algorithm can be tuned in the interface. If needed, segmented spots can be manually edited (deleted or added) thanks to napari Point layer tools and customized shortcuts (Supplementary 2.5, available as supplementary data at *Bioinformatics* online). Cell contours were only marked apically. To assign mRNA molecules to their corresponding cells, we implemented several strategies for an automatic assignment of 3D spots to their cell defined by their 2D apical area. The most obvious assignment method is the Z-projection of spots to the cell apical segmentation. We also propose an assignment based on the closest nucleus, by mixing the two approaches depending on the depth of each spot to the apical surface, by defining a convex hull of the volume of the cell or iteratively by using previous RNA assignments (see comparison of approaches in Supplementary 2.5.2, available as supplementary data at *Bioinformatics* online). In a dense tissue context, manual correction of automatic RNA assignment is necessary to measure correctly the cell genetic profile (Mitchel *et al.* 2025). However, it is also a quite tedious task. We put extra effort in proposing convenient tools for manual correction of RNA assignments based on frequent feedback from the biologists (Supplementary 2.5.2, available as supplementary data at *Bioinformatics* online).

## 3 Additional features

To reduce the number of channels required experimentally when combining IHC and/or DNA/RNA labeling, we have incorporated a tool in FishFeats that permits the computational separation of nuclear staining from an apical junction marker if both are co-stained within the same channel. FishFeats can create two virtual channels containing each biological signal, either based on morphological filters or on a home-made deep learning solution. The latter solution, called SepaNet, was implemented with a U-Net-like architecture, that we separated in two output upsampling parts after the latent space calculation (Supplementary 2.8, available as supplementary data at *Bioinformatics* online). While our neural network is specific to junction-nuclei separation, it could be easily retrained to separate other structures. Recently, other deep learning architectures have also been proposed to implement similar separations (Jin *et al.* 2024, Ashesh *et al.* 2025), which might be interesting to test on our images in future developments.

We propose the option to measure fluorescent intensities in the cytoplasm using a user-defined volume below the apical plane of the cell or in the nuclei following their segmentation (Fig. 1B). Importantly we also added the possibility to manually classify the segmented cells into user-defined categories (e.g. “PCNA-positive cell”). These classifications can be automatically pre-filled from the intensity of any channel (Supplementary 2.9, available as supplementary data at *Bioinformatics* online; Fig. 1B) and/or manually based on the user expertise. Here the user simply clicks on cells to assign them identities. Finally, all these features (cell/nucleus morphologies, transcriptomics content, cytoplasmic or nuclear intensity measures, classification) are joined in a single cell-by-cell table (Fig. 1A), ready for downstream analysis. We added an option in FishFeats to directly perform hierarchical clustering of the cells from that table (Supplementary 2.9, available as supplementary data at *Bioinformatics* online).

## 4 Conclusion

Many powerful image analysis tools are available for a given task. However, for a non-expert user, identifying the right tools, installing and integrating their results together can be quite challenging. The gap between the development of the tools and the accessibility to the user is further increasing with the success of deep learning solutions. Moreover, most analyses require manual corrections, as well as the creation of ground truth datasets to train new deep learning algorithms. There is thus a strong need for more accessible tools in the bioimage analysis field (Carpenter, Kamentsky and Eliceiri 2012, Schlaeppi *et al.* 2022, Soltwedel and Haase 2024, Pylvänäinen *et al.* 2025). We initially developed FishFeats to internally deploy a user-friendly tool for our specific multimodal spatial analyses of tissues at the single-cell level, and are now proposing this development as a flexible and accessible tool to the community.

## 5 Limitations and future developments

The main limitation in the user-friendliness of our plugin is its installation, which requires the creation of a Python virtual environment and the dependencies on multiple libraries. To address this, we have extensively documented the installation steps and proposed alternatively to take advantage of the bundle-app installation mode of napari, a user-friendly procedure for both the installation of the virtual environment and napari.

As our biological applications are focused on epithelial-like cells, we chose to base our pipeline on a 2.5D scheme, segmenting the cells apically from a 2D projection and estimating their Z-position. This limits the application of FishFeats to cells that have a sheet-like organization. Moreover, the 2D projection could induce errors in some measurements, e.g. cell surface area, in regions where a tissue is highly curved. In this scenario, we advise to couple the analysis with solutions such as DeProj (Herbert *et al.* 2021). Finally, 3D nuclear segmentation remains a bottleneck: existing solutions are, in general, not precise enough, or computationally expensive on large images, and manual correction is more complicated in 3D. We plan to expand FishFeats with forthcoming developments on 3D nuclear segmentation or correction, incorporating emerging tools such as nnInteractive (Isensee *et al.* 2025).

Overall, we expect FishFeats to keep evolving as new community needs arise. Our goal is to ensure long-term maintenance and usability of the pipeline, providing a reliable framework for accessible bioimage analysis in the years to come.

## Supplementary Material

btag105_Supplementary_Data
